# Assessing alkali activation of waste stone wool from greenhouses combined with direct foaming or granulation to obtain recycled plant substrate

**DOI:** 10.12688/openreseurope.17101.1

**Published:** 2024-02-12

**Authors:** Karine Goulart de Oliveira, Soile Jokipii-Lukkari, Tero Luukkonen

**Affiliations:** 1 Fibre and Particle Engineering Research Unit, University of Oulu, Oulu, Northern Ostrobothnia, 90014, Finland; 2 Department of Chemical Engineering, Universidade Federal de Santa Catarina, Florianópolis, State of Santa Catarina, 88040-900, Brazil; 3 Ecology and Genetics Research Unit, University of Oulu, Oulu, Northern Ostrobothnia, 90014, Finland

**Keywords:** agriculture, circular economy, geopolymers, greenhouse cultivation, stone wool

## Abstract

**Background:**

Stone wool is commonly used as a plant substrate in soilless cultivation and discarded after one growing season. Stone wool waste is difficult to recycle, and thus it is typically landfilled. Alkali-activation of stone wool (i.e., milling and mixing with an alkaline solution) has been shown to be a feasible way to upcycle this waste fraction into, for example, construction products. In this study, the aim was to develop recycled plant substrate from stone wool waste from greenhouses via alkali activation.

**Methods:**

Waste stone wool from greenhouses was characterized by X-ray fluorescence (XRF) and mixed with sodium silicate solution either directly or after ball milling. The alkali-activation process was combined with the addition of H _2_O _2_, pre-made foam, or granulation to obtain suitable porous material for the plant substrate application. Preliminary greenhouse cultivation experiments of pea ( *Pisum sativum*) were conducted with alkali-activated stone wool mixed with peat (a weight ratio of 1:1) and fertility analysis of the mixture were conducted.

**Results:**

The results indicated that the most feasible production method was to use ball-milled stone wool and to combine alkali activation with granulation. The obtained granules could reach 2.7 MPa as compressive strength while the other methods resulted in very fragile material. The preliminary greenhouse cultivation experiments revealed that there were significant levels of nutrients (Ca, P, K, and S) and alkalinity leached from the granules which hindered the growth of pea. The high P and S amounts were also confirmed by the XRF results of stone wool.

**Conclusions:**

It can be concluded that the developed granules did not function well as a plant substrate for pea but could enable the re-utilization of the nutrients contained in the greenhouse stone wool waste. Moreover, their application to acidic sulfate soils could be feasible as it would utilize the alkalinity of granules.

## Introduction

The development of alkali-activated materials, including geopolymers, from alkaline and aluminosilicate side streams has become an active research topic to provide upcycling prospects for secondary raw materials ^1, 2^. The interest in the use of alkali-activated materials as a low-CO _2_ alternative binder for Portland cement in concrete has increased prominently in the last few decades with emerging commercial implementations ^3^. Alkali-activated materials are prepared by a process in which aluminosilicate precursor (partially) dissolves in an alkaline medium (e.g., aqueous sodium silicate solution) at (near-)ambient conditions (i.e., normal pressure and temperature of 20–80 °C) and forms subsequently new mineral phases. The main phases range from zeolite-like aluminosilicate networks to tobermorite-like aluminosilicate chains depending on the composition of the precursor, especially its calcium content ^4^.

Mineral wool (i.e., stone or glass wool) consist of synthetic vitreous fibers prepared by melting of mainly basalt and diabase in the case of stone wool while glass wool is prepared by melting sand, limestone, soda, and borax ^5^. After that, the melted material is fiberized by a spinner wheel or spinning machine ^5^. Mineral wool is commonly used for thermal insulation and acoustic panels in buildings ^6^. Thus, construction and demolition waste contains significant amounts of mineral wool, with an estimated annual generation of 2.3 Mt in Europe alone ^6^. Mineral wool is difficult to recycle due to its low density, and thus it remains largely landfilled ^6^. One prospect for the utilization of mineral wool waste is to use it as a precursor for alkali-activated materials to develop construction products ^7, 8^, which was the main topic of the Horizon 2020 project WOOL2LOOP (Mineral wool waste back to loop with advanced sorting, pre-treatment, and alkali activation) ^9^.

Stone wool (sometimes referred to as rock wool) is also widely used as a plant substrate in greenhouses in soilless cultivation such as hydroponic systems since the 1970s ^10, 11^. There, some useful attributes of stone wool include enabling an easy recirculation of water, decrease of water usage, reuse of the nutrients, and absence of soil diseases ^11^. However, it is a common practice to dispose of the stone wool after just one growing cycle, which leads to an estimated accumulation of 150 m ^3^ of stone wool waste per 1 ha of growing area ^12^. In a case study involving cucumber cultivation, the direct reuse of stone wool was observed to reduce some morphological parameters such as leaf area or number of leaves per week already on the second cultivation cycle ^13^. Thus, there is a clear need to find post-life solutions for the stone wool substrate waste.

Due to their composition and amorphous structure, both glass wool and stone wool are suitable precursors for alkali activation ^14^. Alkali-activated mineral wools can be utilized, for example, as binder for concrete, preparation of solid foams, or façade panels ^15– 17^. One challenge with mineral wools in this context is organic resin, such as phenol formaldehyde, added to the mineral wool products during their manufacturing, which may interfere with the alkali-activation reactions ^18^. However, the fact that stone wool intended for green houses does not contain organic additives (in order to be water-absorbing) similarly as the mineral wools used in construction, makes it a highly potential precursor ^11, 19^.

The aim of this work is to provide a potential afterlife solution for stone wool waste from greenhouses by recycling it into alkali-activated plant substrate material. In this study, two production methods were compared: production of alkali-activated foams or granules. The most feasible methodology revolves around the combined alkali activation–granulation process in which spherical alkali-activated granules are produced by placing the precursor in a granulator and slowly adding the alkali-activator solution while the granulator is running. This results in the formation of granules and subsequent dissolution of the precursor forming eventually the alkali-activated phases at (near-)ambient temperature ^20^. In this study, the methodology to granulate mineral wool waste was developed, the obtained granules were characterized for their mechanical and physico-chemical proprieties, and a preliminarily assessment of their suitability as a plant substrate via soil fertility study and growth experiment of pea ( *Pisum sativum*) was conducted.

## Methods

### Stone wool waste

**Figure 1.**
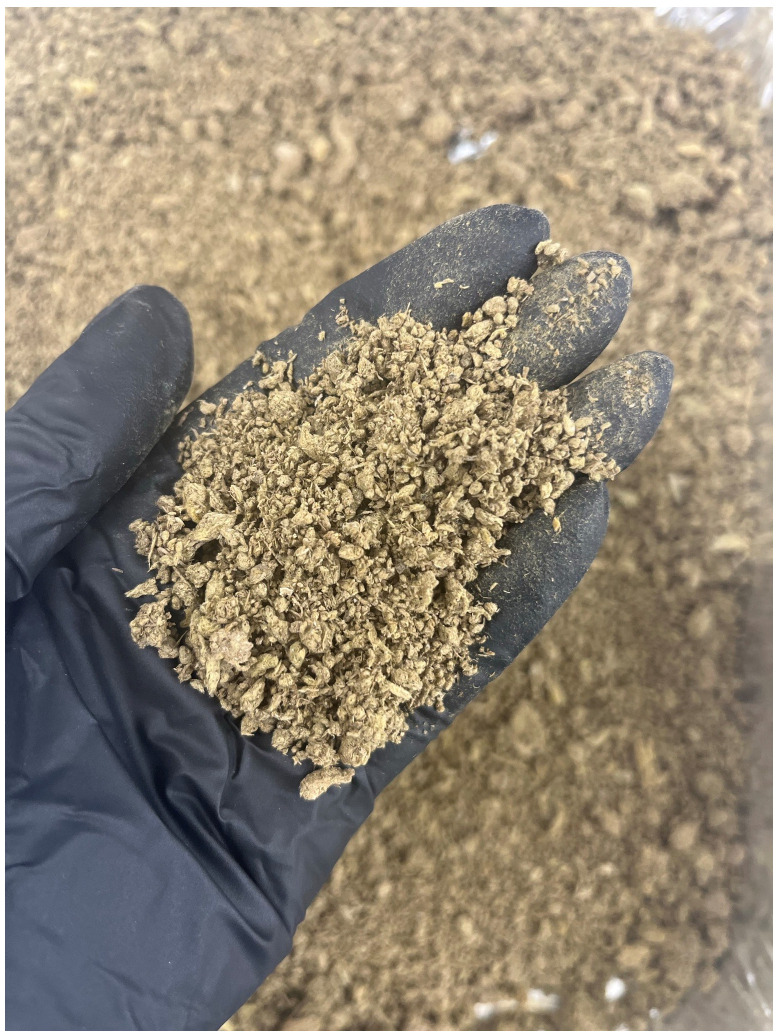
As-received stone wool waste from greenhouses.

Stone wool waste used for one season in greenhouses was obtained from Netherlands. The stone wool waste studied in the present study is managed as follows: at the end of the growing season, after the recovery from the professional growers, a recycling company conducts the separation of stone wool from plastic and organic matter fractions. The granules of stone wool are then re-sent to the material provider for subsequent waste management, such as landfilling. The raw stone wool waste ( Figure 1) contained 45% of dry matter, from which 90% corresponded to stone wool, 10% to organic matter, and 0.014% to polyethylene plastics. Plastic residues were introduced to the stone wool waste during their use in greenhouses. The chemical composition of stone wool waste as determined by X-ray fluorescence (XRF, PANalytical Omnian Axios mAX, Malvern Panalytical with the Omnian software package, analysis was conducted for pressed powder) and reported as oxides is shown in Table 1 in comparison to a typical stone wool composition according to Yliniemi *et al*. ^18^. All other element quantities are within the typical ranges except Si which is slightly lower and P and S which are clearly higher. P and S are likely introduced by the fertilizers used during the growing cycle. Also, loss on ignition (LOI) at 525 °C is higher than with the typical composition likely indicating the presence of organic matter from plants. In addition, LOI at 950 °C was determined (not shown in Table 1) but no further mass loss (< 0.1 weight-%) was observed indicating that no carbonates or other minerals decomposing at that temperature were present.

**Table 1.** The chemical composition and loss on ignition (LOI) of stone wool waste from greenhouse in comparison to a typical stone wool composition according to the literature.

Sample name	CaO	SiO _2_	Al _2_O _3_	Fe _2_O _3_	Na _2_O	K _2_O	MgO	P _2_O _5_	TiO _2_	SO _3_	Cl	LOI (525 °C)
Stone wool waste from greenhouse	17.68	32.98	13.63	6.68	2.13	0.91	7.57	2.78	1.67	1.07	0.17	9.8
Typical stone wool ^a^	14.43– 40.10	39.05– 54.00	7.00– 24.00	5.48– 13.22	1.38– 6.20	0.13– 3.80	2.00– 13.00	0.00– 0.80	0.50– 3.06	0.06– 0.23	0.04– 0.19	1.40– 7.40

a = range of compositions in typical stone wool according to Yliniemi *et al*. ^18^.

### Alkali-activation of stone wool waste

**Figure 2.**
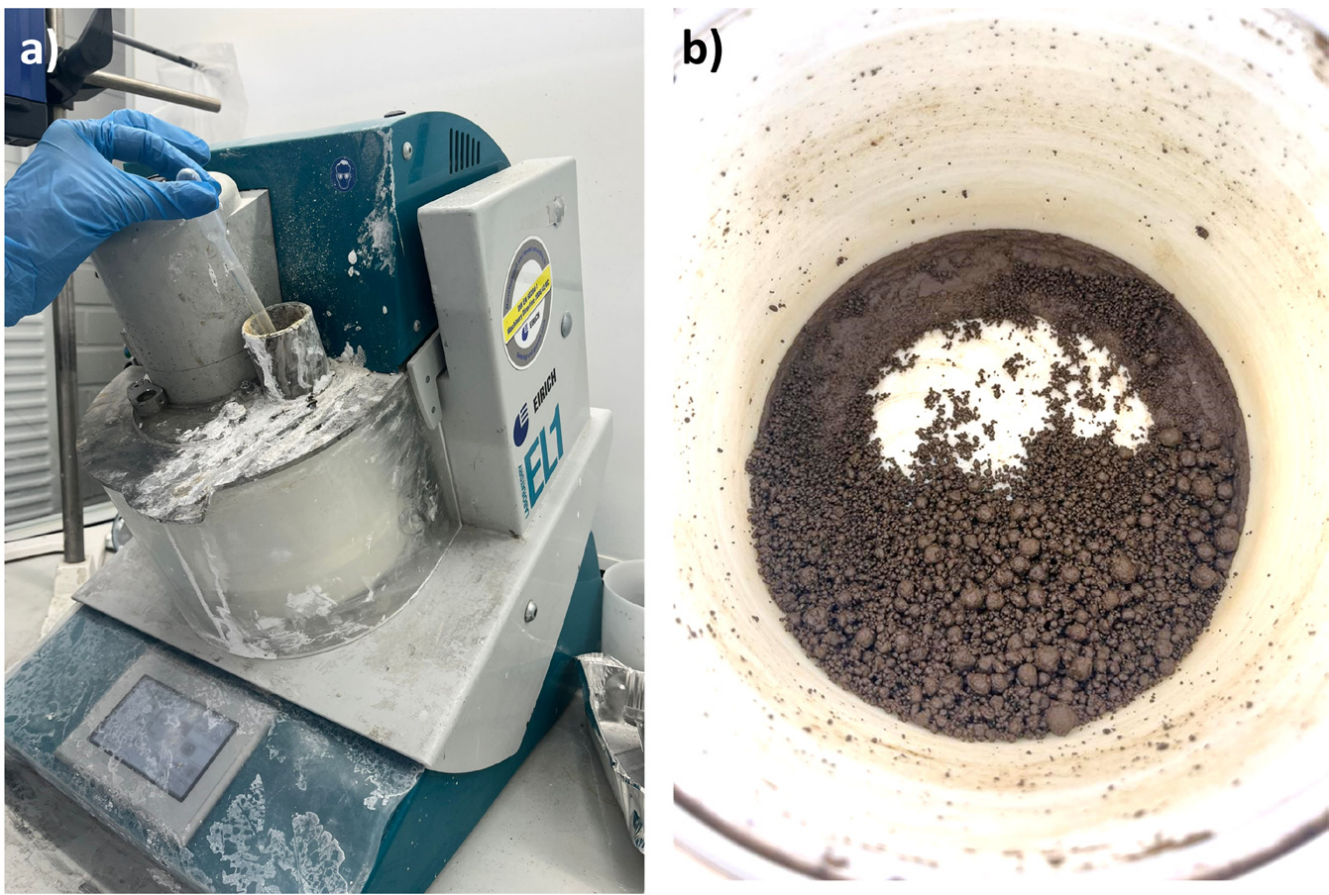
**a**) High-shear granulator used in the experiments, **b**) appearance of the granules before sieving and curing.

Since stone wool waste arrived with a high water content (55 weight-%), it was dried in an oven for 48 hours at 60 °C. The dried stone wool was ground with a jar mill Topfroller (Germatec) for 2 h using the maximum speed (460 rpm). The particle size distribution was quantified with a laser diffraction particle size analyzer (LS 13 320, Beckman Coulter) by dispersing the sample in 0.5 weight-% isopropyl alcohol. The d _10_, d _50_, and d _90_ values were 2.8, 16.8, and 70.3 µm, respectively, after milling. Figure 2 shows the appearance of stone wool waste after drying and milling.

The alkali-activator solution used in the experiments described below was prepared by mixing 5 M NaOH solution (VWR Chemicals, Sweden) and sodium silicate solution (Betol 52T, Woellner, Germany; containing 14.7 wt% Na _2_O, 30.3 wt% SiO _2_, and 55 wt% H _2_O) in a weight ratio of 1:1.

***Preparation of alkali-activated stone wool foams.*** The first batch of alkali-activated stone wool foam was prepared by combining dried stone wool without milling with alkali-activator solution in a weight ratio of 10:8 and mixing 15 min with a planetary mixer. Next, foam (30 weight-% of the mixture) was introduced and mixing was continued for 10 min. The foam was generated with an EABASSOC Junior Foam Generator using EABASSOC Foaming Agent diluted to 3 weight-% with water. The aim of this experiment was to assess whether the stone wool waste could be utilized without any milling as a pretreatment.

The second batch of alkali-activated stone wool foam was prepared similarly as above but by using stone wool waste milled for 2 h as the precursor and using unmilled stone wool as a fiber additive. The aim of the fiber addition was to improve the mechanical strength of the foam. The foam and unmilled stone wool were added as 20 and 5 weight-% of the mixture, respectively.

Finally, a third experiment was conducted using again the 2-h milled stone wool with alkali-activator solution, mixed similarly as above, but this time using 30% hydrogen peroxide (VWR) as a blowing agent. Hydrogen peroxide was added as 1.75, 2.50, or 3.50 weight-% of the mixture.

In the foam experiments, the mixture was cast in 160 × 40 × 40 mm-sized steel molds, placed in a plastic bag, and cured at 60 °C for 24 h. After that, the samples were stored at ambient conditions.

***Preparation of alkali-activated stone wool granules.*** The granules were produced by placing the 2-h milled stone wool in the drum of a high-shear granulator (Eirich EL1) and the alkali-activator solution was slowly added into the moving drum until the weight ratio of alkali-activator and stone wool of 0.45 was reached. Mixing in the granulator was continued for 5 minutes, granules between sizes of 1 to 4 mm were separated by sieving, added into a plastic bag, and placed in an oven at 60° C for 24 hours.

### Characterization methods of alkali-activated stone wool samples

The open, closed, and total porosities were obtained by measuring the density of the material using a helium pycnometer (AccuPyc II 1340, Micromeritics). The geometric density ( *ρ _g_*, g/cm ^3^), also called bulk density, was determined by measurement of the mean diameter of the granules using a digital caliper and their mass. The apparent density ( *ρ*_*a*_, g/cm ^3^) was obtained by helium pycnometry of the entire granules and the true density ( *ρ*_*t*_, g/cm ^3^) was measured by the same method from pulverized granules. The porosity was calculated according to Equation 1– Equation 3.

$$\text{Open}\;\text{porosity}=\frac{\rho_{a}-\rho_{g}}{\rho_{a}}x100\;(1)$$

$$\text{Total}\;\text{porosity}=\frac{\rho_{t}-\rho_{g}}{\rho_{t}}x100\;(2)$$

Closedporosity=Totalporosity−Openporosity(3)

To determine the mechanical strength of the granules, the material was subjected to a uniaxial compression test with a universal testing machine Zwick Roell Z010, with a constant crosshead speed of 0.5 mm/min. The compressive strength was calculated with Equation 4, where *F* is the maximum load (N) at break applied in the material, that is dependent on the radius, *R*, or diameter, *D*, since the structure has spherical shape. The load factor 1.4 corresponds to the peak stress approximate when the load is distributed over 2θ or ~10 ° as described by ^21^.

$$\sigma_{\theta }=1.4\frac{F}{2\pi R^{2}}=0.9\frac{F}{D^{2}}\;(4)$$

Specific surface area was determined with the Brunauer–Emmett–Teller (BET) isotherm. The samples were degassed at 110 °C for 16 h. After that, the analysis was performed by N _2_ adsorption at liquid nitrogen temperature with the ASAP 2020 (Micromeritics) instrument. For the calculation of total pore volume, average pore diameter, and pore size distribution, the Barrett–Joyner–Halenda (BJH) method was used.

The morphology and composition of the samples was analyzed through optical microscopy (STEMI 2000-C, Carl Zeiss AG) and scanning electron microscopy combined with energy-dispersive spectroscope (SEM, Zeiss Ultra Plus).

Raw data of alkali-activated stone wool characterization is available in ^22^

### Greenhouse cultivation experiment

The granulated stone wool was used in a preliminary greenhouse experiment to investigate the plant germination and growth using pea ( *Pisum sativum* 'Kelvedon Wonder', Nelson Garden) seeds. Before the experiment, the granules were neutralized by immersing them in an acetic acid solution (0.1 M) for 30 min, rinsing with deionized water and repeating this until the pH of the rinsing water reached neutrality.

In the greenhouse experiments, one seed per pot (7 × 7 × 6.5 cm) was sown in peat (Kekkilä FPM 420 W F6 R8039; controls) or in mixture of stone wool granules and peat (weight ratio of 1:1). A total of 56 seeds were sown in both groups. Water was supplied at a few days’ intervals and no additional fertilizers were given. The photoperiod was maintained for 12 h with Luminatec Areal 300-150W-120-0104 LED lamps. The average temperature was 25 °C during the day and 20 °C at night. The experiment started on January 17, 2023 and continued until February 8, 2023. The germination rate and shoot and root lengths were recorded in the end of the experiment. After examination of the normal distribution of the data by Shapiro–Wilk’s test, the shoot and root lengths between control and treatment groups were compared using Wilcoxon signed-rank test in RStudio (version 1.3.1093).

The fertility of the stone wool granules–peat mixture was analyzed by determining its exchangeable and easily soluble nutrient amounts (P, Ca, K, Mg, Na, S, B, Cu, Mn, and Zn) as well as pH and conductivity. In short, the samples were air-dried and extracted with a 0.5 M ammonium acetate and 0.5 M acetic acid solution (pH = 4.65) for 1 h using a 1:10 (V/V) mineral wool granules–peat mixture to solution ratio ^23^. The elemental analyses were conducted with an optical emission spectrometer (Thermo Electron IRIS Intrepid II XDL Duo) according to the standard method ^24^.

## 3 Results and discussion

### 3.1 Trials with alkali-activated stone wool foams

**Figure 3.**
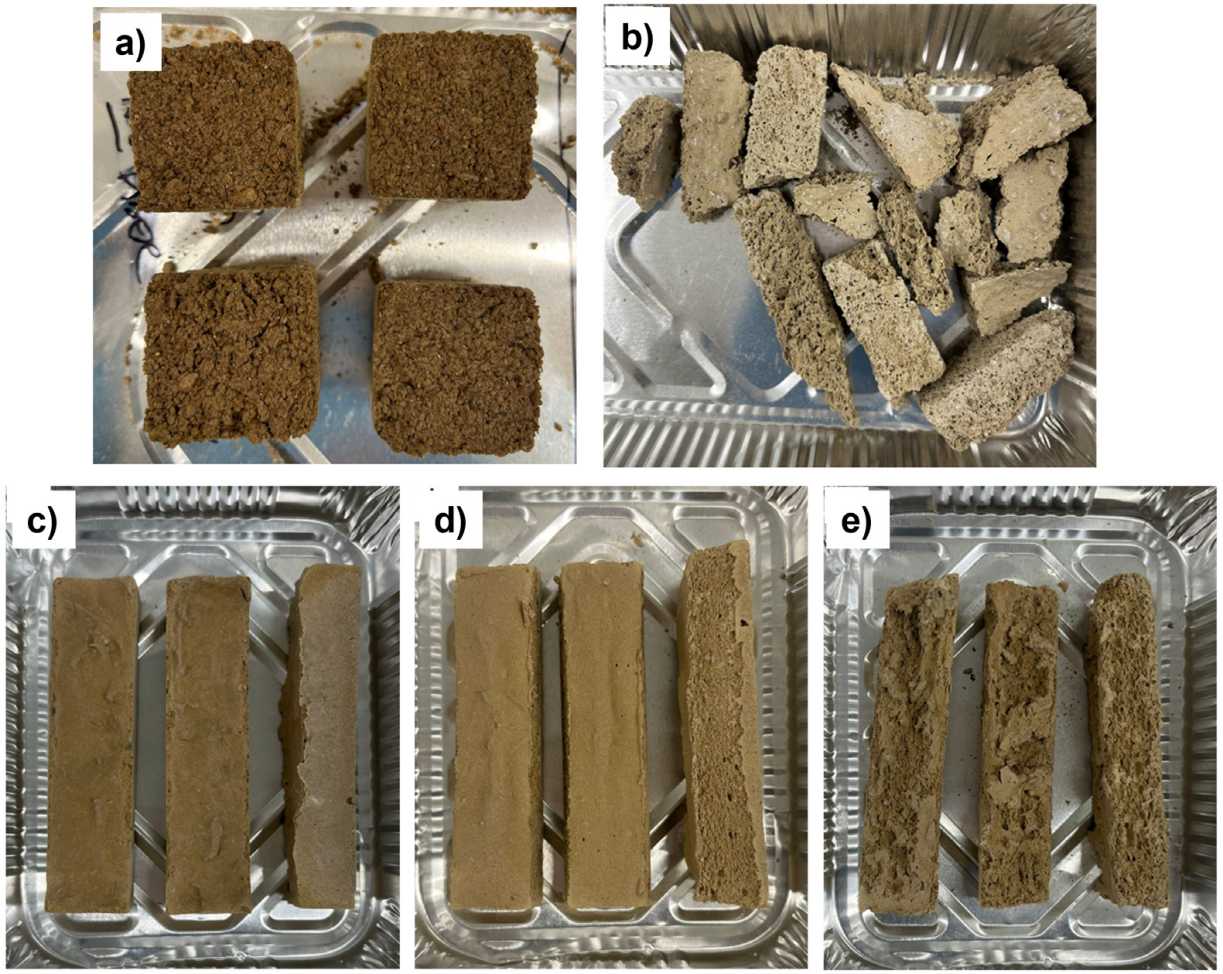
Appearance of the alkali-activated stone wool samples prepared with **a**) unmilled stone wool and foam, **b**) milled stone wool with unmilled stone wool and foam, **c**) milled stone wool with 1.75 weight-% hydrogen peroxide, **d**) milled stone wool with 2.50 weight-% hydrogen peroxide, and **e**) milled stone wool with 3.50 weight-% hydrogen peroxide.

The alkali-activated stone wool foam samples prepared with either pre-generated foam or hydrogen peroxide proved to be unsatisfactory in terms of their mechanical strength as they were too brittle and fragile to be handled in the context of greenhouse cultivation ( Figure 3). Their compressive strength was too low to be determined.

When using un-milled stone wool ( Figure 3a), its dissolution to the alkali-activator solution likely remained low due to its too large particle size. The bulk density of the foam material containing un-milled wool was approx. 0.5 g/cm ^3^ which can be compared to the study of Pavlin *et al*. ^16^ in which they prepared alkali-activated stone wool foams by using hydrogen peroxide: the bending strength of their foams approached 0 MPa as the bulk density was less than 0.6 g/cm ^3^ which in agreement with our result.

When using the milled stone wool with either pre-generated foam or hydrogen peroxide ( Figure 3b-e), the obtained alkali-activated foams remained still too fragile. The bulk density of the foam in Figure 3b was approx. 0.5 g/cm ^3^ while hydrogen peroxide-containing samples had bulk densities of 1.0–1.1 g/cm ^3^. The milled mineral wool had an approximately comparable particle size distribution to that of Pavlin *et al*. ^16^ and they were able to use it successfully for the preparation of alkali-activated foams with sufficient mechanical strength. A possible reason for the low mechanical strength in the present study of the hydrogen peroxide-containing samples could be the high viscosity of the paste which required additional water and subsequently diluting the alkali-activator solution. When using the pre-generated foam, the foam itself introduced extra water to the system. Thus, the direct foaming or addition of pre-generated foam were ruled out as plant substrate preparation methods and the samples were not characterized further in the present study. Moreover, the foaming process has been criticized to be likely difficult to up-scale to an industrial level ^25^.

### 3.2 Alkali-activated stone wool granules

**Figure 4.**
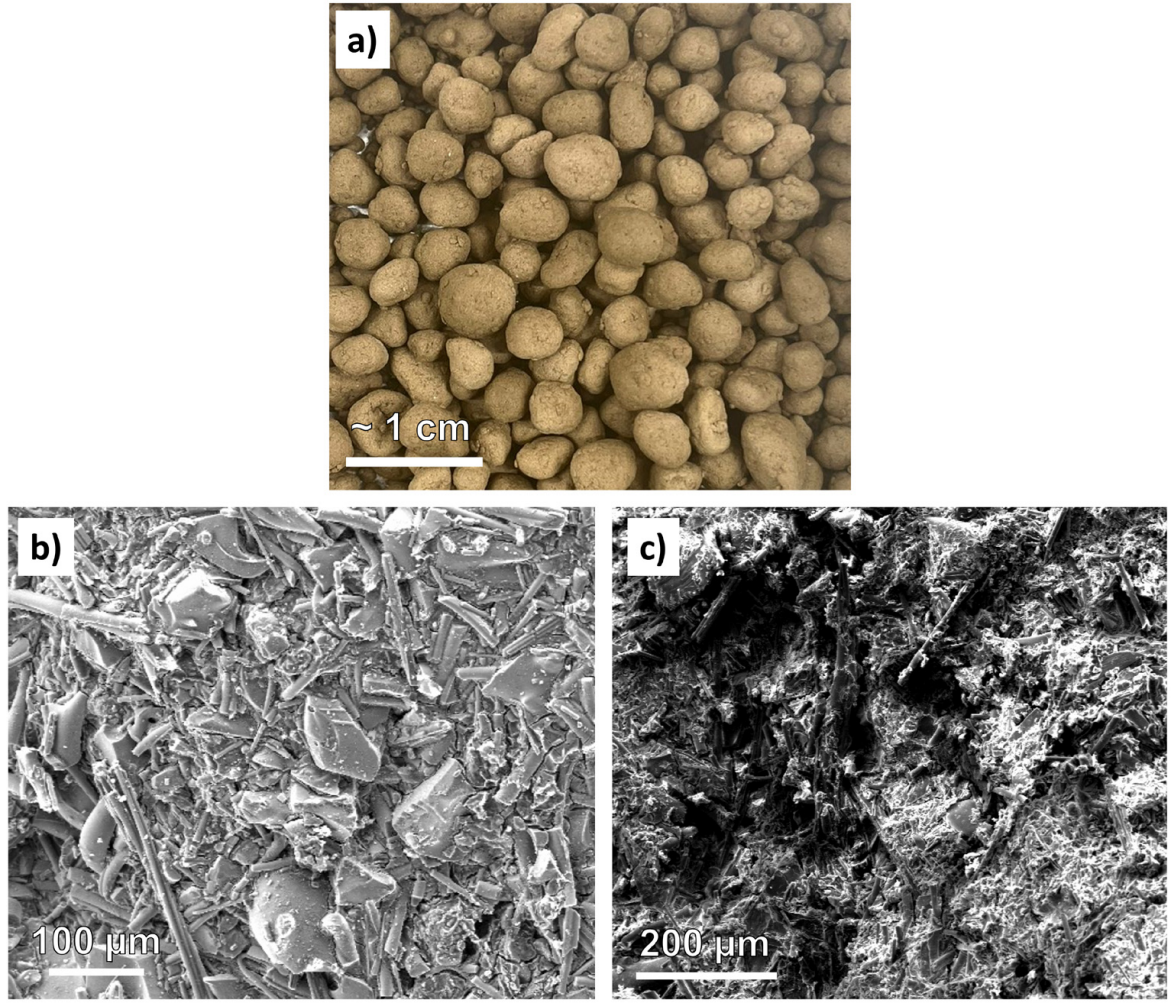
**a**) Appearance of the alkali-activated stone wool granules after curing. Micrographs of **b**) the granule surface and **c**) cross-section.

The granulation of milled stone wool waste resulted in robust and easily handleable material ( Figure 4a) that could be potentially utilized similarly as light-weight expanded clay aggregate (LECA) as a plant growing medium ^26^. The alkali-activated stone wool granules had sufficient compressive strength of 2.7 ± 0.7 MPa which is comparable to the LECA granules of approximately similar size (5–6 mm) ^27^. Their total, open, and closed porosities were 27.8, 25.7, and 2.2%, respectively. The electron microscope analyses indicated that the interior of the granules ( Figure 4c) contained well-reacted phases with only a few unreacted stone wool fiber fragments visible. On the other hand, the surface of the granule ( Figure 4b) was covered with largely unreacted stone wool fibers with length varying from tens to hundreds of µm. Moreover, there were also approx. 100 µm-sized particulates attached to the surface of the granules. EDX analysis revealed that that these particles were calcium phosphate (with P weight-% of approximately 17%). In the interior of the granules such particulates were not present.

### 3.3 Assessment of alkali-activated stone wool as plant substrate

**Figure 5.**
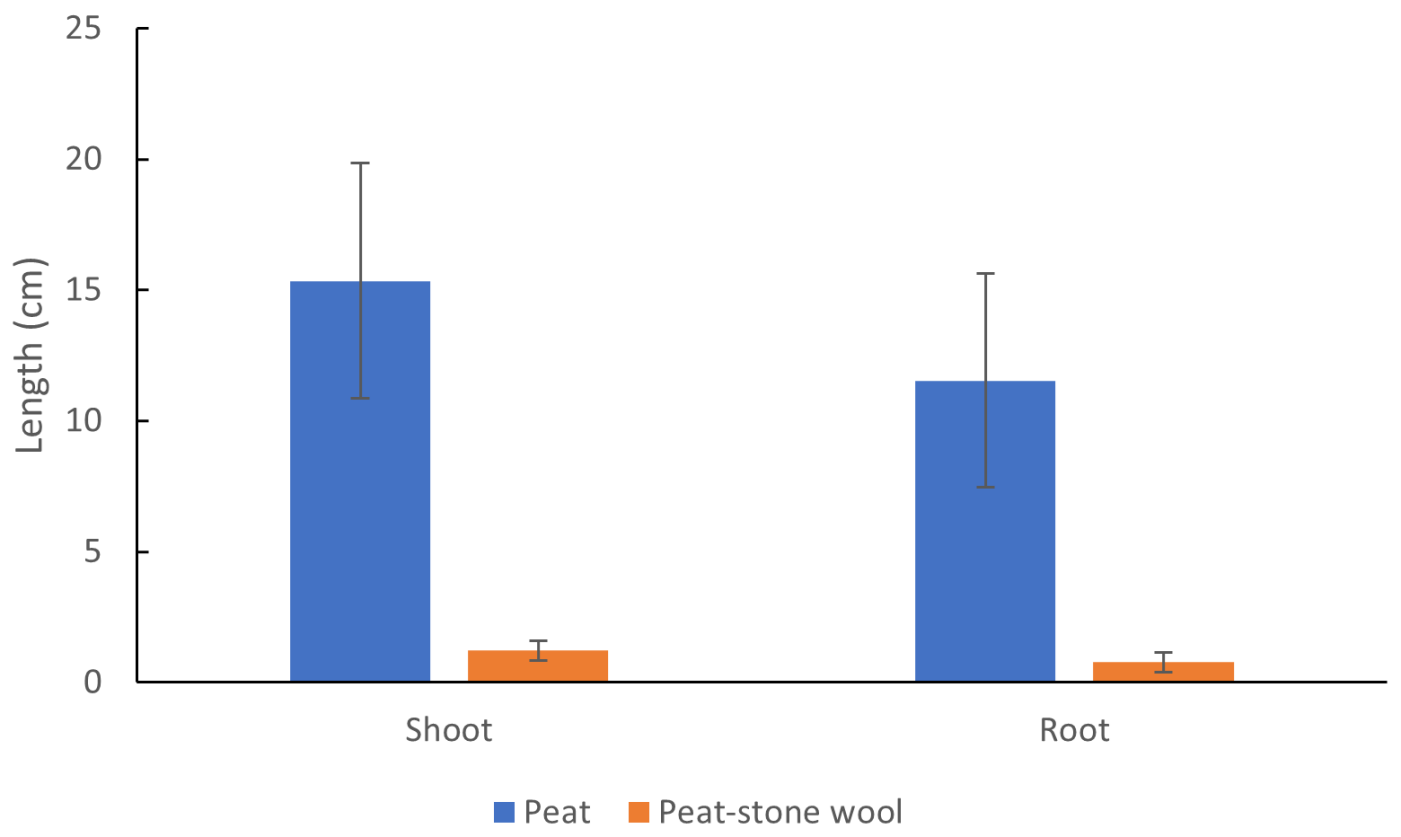
The shoot and root lengths of peas grown in peat or mixed recycled stone wool and peat (1:1) after 22 days of greenhouse cultivation. Values are means ± standard deviation. Stars above the columns denote a statistically significant ( *P* < 0.001) difference between the means of control and treatment groups.

**Figure 6.**
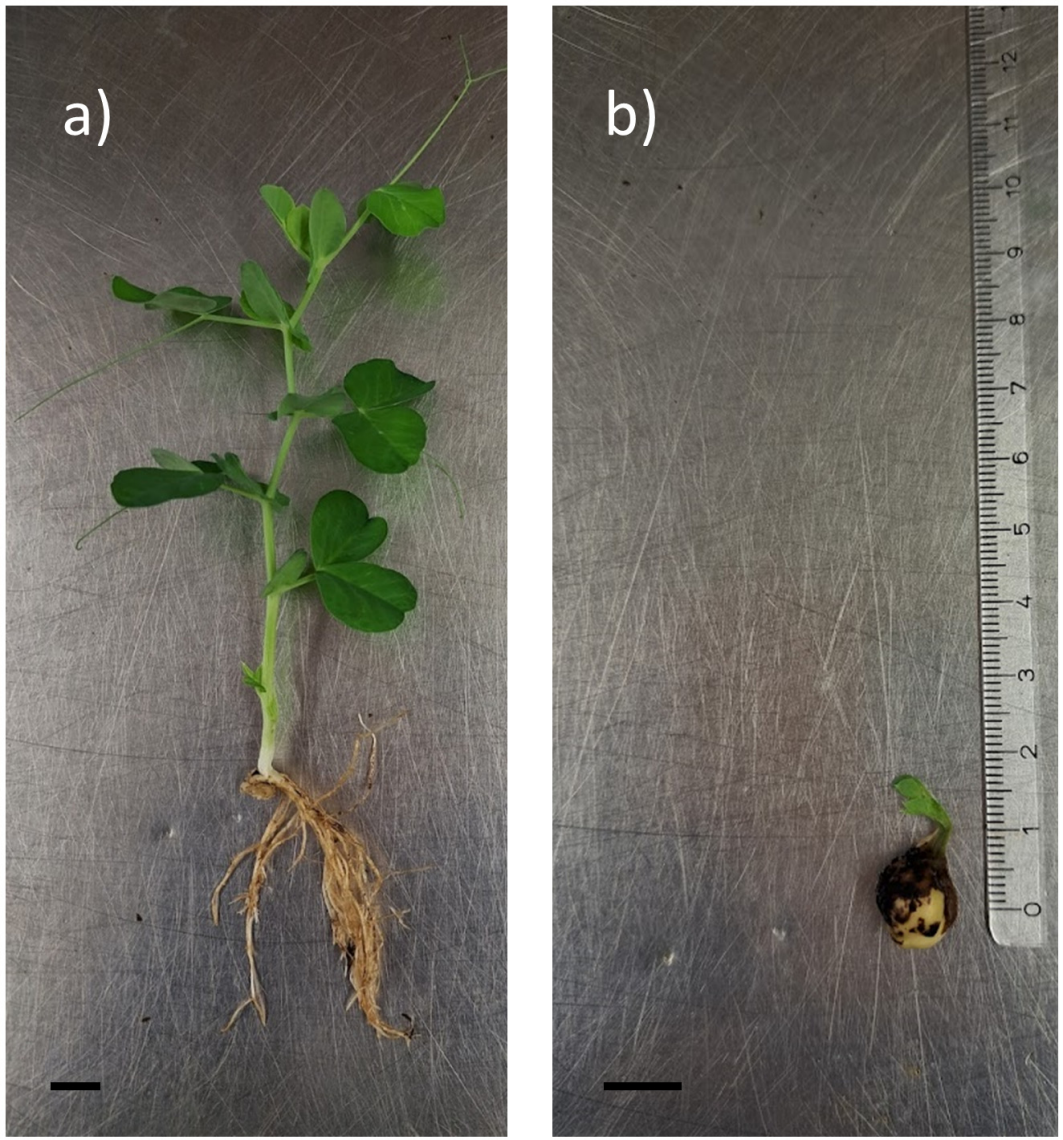
The growth habit of peas grown in ( **a**) peat or ( **b**) mixed stone wool granules and peat (1:1) after 22 days of greenhouse cultivation. The scale bar represents 1 cm.

After 22 days of greenhouse cultivation, the germination percentage of control seeds grown in peat was 98.2% whereas only 73.2% seeds cultivated in the mixture of recycled stone wool and peat had germinated. The shoot and root lengths of controls were 12.7 and 14.9-fold in comparison to treatment group, respectively ( Figure 5). The growth habit of the seedlings grown on mixed stone wool and peat was twisty and down to soil ( Figure 6).

To understand the reasons for the poor growth on the peat-stone wool mixture, soil fertility analyses were conducted ( Table 2). The results showed that there was significant level of nutrients leached from the granules making the many of the nutrient levels “suspiciously high” according to the Finnish guideline values for agricultural soils ^28^. The results implicate that the granules developed in the present study could be better suited to be used as a fertilizer product instead of a plant substrate, and thus their amount in soil should be much lower than in the greenhouse cultivation experiment (in which the peat: stone wool were used as 1:1 mixture). Perhaps the primary reason for the poor growth was the high pH of the soil (9.4) since the optimum pH for pea is approximately 6.1–7.1 ^28^. It has been shown earlier that pH of alkali-activated materials can be effectively neutralized with dilute acid solutions ^29^ as done in the present study. However, if the alkali-activated material has a high Ca content, the leaching of alkalinity tends to continue over time as was also observed in the present study. The exact mechanism for this continued leaching is unclear. However, this property could be utilized in the agriculture since, for instance, in Finland acidic sulfate soils require pH increase which is conventionally done by applying agricultural lime to the soils. Thus, the alkali-activated stone wool granules could possibly be used as a source of nutrients and alkalinity.

**Table 2.** Results of the fertility analysis of the alkali-activated stone wool granule–peat mixture and their interpretation according to the Finnish guideline values for agricultural soils ^28^.

Parameter	Result	Interpretation
Conductivity [mS/cm]	30.7	-
pH	9.4	Suspiciously high
Ca [mg/L]	10000	Suspiciously high
P [mg/L]	340	Suspiciously high
K [mg/L]	1200	Suspiciously high
Mg [mg/L]	1000	High
Na [mg/L]	9700	Good
S [mg/L]	350	Suspiciously high
B [mg/L]	6.7	Suspiciously high
Cu [mg/L]	7.2	Satisfactory
Mn [mg/L]	15	Tolerable
Zn [mg/L]	46	High

## 4 Conclusions

Stone wool used as a plant cultivation media in greenhouses was studied as a precursor for alkali-activated materials to obtain recycled plant substrate. Two main methods were studied: direct foaming or premade foam addition and granulation combined with alkali activation. Direct foaming or premade foam addition proved to be unfeasible as the obtained material was too fragile to be handled in the greenhouse environment. Granulation, on the other hand, produced robust material whose mechanical strength is comparable to the commonly used LECA granules. Preliminary greenhouse cultivation experiments indicated that when the granules were used as 1:1 mixture with peat and used to grow pea, the growth was inhibited. Soil fertility analysis indicated that there was a significant level of nutrients as well as alkalinity leaching from the granule–peat mixture. Thus, the developed granules could be more suited as a combined fertilizer and soil pH adjustment product. However, more research would be needed to determine for example the nutrient release kinetics and validate the greenhouse cultivation using lower doses of granules mixed with peat or other types of soil.

## Ethics and consent

Ethical approval and consent were not required.

## Data Availability

Fairdata: Underlying data for ‘Assessing alkali activation of waste stone wool from greenhouses combined with direct foaming or granulation to obtain recycled plant substrate’, https://doi.org/10.23729/7658573a-b677-4f3b-a71a-796044d4e3f2 ^22^ This project contains the following underlying data: Compressive strength of alkali-activated stone wool samples Density of alkali-activated stone wool samples Loss on ignition (LOI) of alkali-actvated stone wool samples Data are available under the terms of the Creative Commons Attribution 4.0 International license (CC-BY 4.0)
