# Supplementary material for: Assessing alkali activation of waste stone wool from greenhouses combined with direct foaming or granulation to obtain recycled plant substrate
Source: Open Res Eur. 2024 Feb 12;4:26. [Version 1] doi: 10.12688/openreseurope.17101.1 (PMC11036038; doi:10.12688/openreseurope.17101.1)
Supplement: Peer review history

# Reviewer report: Reviewer response for version 1

**DOI:** 10.21956/openreseurope.18482.r38309

**Published:** 2024-04-22

**Authors:** Muhammad Riaz Ahmad

**Affiliations:** 1 The Hong Kong Polytechnic University, Hong Kong, Hong Kong

1. The article lacks a comprehensive introduction that provides background information on the importance and challenges of finding suitable plant substrates for greenhouse cultivation. It would be helpful to include a brief overview of the current methods and materials used in the industry and the motivation for exploring alkali-activated stone wool as a potential alternative.

 2. Could you provide more information about the characteristics of the alkali-activated stone wool granules, such as their porosity, water retention capacity, and nutrient-holding capacity? These properties play a crucial role in determining their suitability as a plant substrate.

 3. The discussion on the soil fertility analysis results is informative, but it lacks a clear connection to the observed poor growth of the plants. It would be beneficial to discuss how the leaching of nutrients and alkalinity from the granules could directly impact plant growth and potentially inhibit germination and development.

 4. Are there any environmental considerations or sustainability aspects associated with the use of alkali-activated stone wool as a plant substrate? Considering the increasing emphasis on sustainable agriculture, it would be valuable to discuss the potential benefits or drawbacks of this material in terms of resource consumption, waste reduction, and carbon footprint.

Is the study design appropriate and does the work have academic merit?

Partly

Is the work clearly and accurately presented and does it cite the current literature?

Partly

If applicable, is the statistical analysis and its interpretation appropriate?

Partly

Are all the source data underlying the results available to ensure full reproducibility?

Partly

Are the conclusions drawn adequately supported by the results?

Partly

Are sufficient details of methods and analysis provided to allow replication by others?

Partly

Reviewer Expertise:

Alkali Activated Materials, Cement and Concrete

I confirm that I have read this submission and believe that I have an appropriate level of expertise to confirm that it is of an acceptable scientific standard, however I have significant reservations, as outlined above.

# Reviewer report: Reviewer response for version 1

**DOI:** 10.21956/openreseurope.18482.r39426

**Published:** 2024-04-19

**Authors:** Beibei Sun

**Affiliations:** 1 Magnel-Vandepitte Laboratory, Ghent University, Gent, Belgium

To find a post-life solution for stone wool, reduce CO2 emissions, and avoid the economic burden of landfills, the authors innovatively explored the production of alkali-activated stone wool for agriculture applications. From the attempts on 3 types of alkali-activated stone wool foams and 1 alkali-activated stone wool granule, it was found that alkali-activated stone wool granules could be suitable fertilizer products due to qualified compressive strength and fast neutralization speed. The greenhouse cultivation capacity of alkali-activated stone wool granules was quantitively determined and the mechanisms were clarified using advanced techniques. This paper demonstrates a successful exploration. More research can be carried out based on this study in the future to facilitate the utilization of industrial by-products.

Is the study design appropriate and does the work have academic merit?

Yes

Is the work clearly and accurately presented and does it cite the current literature?

Yes

If applicable, is the statistical analysis and its interpretation appropriate?

Yes

Are all the source data underlying the results available to ensure full reproducibility?

Yes

Are the conclusions drawn adequately supported by the results?

Yes

Are sufficient details of methods and analysis provided to allow replication by others?

Yes

Reviewer Expertise:

mix design of alkali-activated material

I confirm that I have read this submission and believe that I have an appropriate level of expertise to confirm that it is of an acceptable scientific standard.

# Reviewer report: Reviewer response for version 1

**DOI:** 10.21956/openreseurope.18482.r38307

**Published:** 2024-03-14

**Authors:** Tao Yang

**Affiliations:** 1 Yancheng Institute of Technology, Yancheng, Jiangsu, China

This is an interesting topic about the alkali-activation of waste stone wool. The alkali-activated waste stone wool foam and granules were produced in this work. The availability of using such material as plant substrate was also evaluated. This work is meaningful for greener recycling of waste stone wool. However, only few formulations and few measurements were performed in this work. The reviewer strongly suggested to perform some tests (mentioned below) to confirm the reaction between waste stone wool and the alkaline activator. The specific comments are listed as follows.

1. The whole methods and testing Section included too much steps. It is suggested to provide one or two schematic diagrams to illustrate the alkali-activation of stone wool and the greenhouse cultivation experiment.
2. The XRD characterization for the stone wool waste should be provided.
3. The main purpose of using alkali activation to treat and use solid waste is to enhance the reactivity of the waste and prepare cementitious materials with certain strength development. However, in this work, the alkali-activated stone wool foam was too brittle and fragile. As to the artificial alkali-activated stone wool granules, the strengths test or other crushing index experiment was not performed. So how can we speculate or assure that the stone wool exhibited reactivity during alkali-activation? Or how can we make sure the alkali-activation is meaningful for recycling the waste stone wool? It is strongly suggested to perform some strengths test. In addition, XRD, FTIR or TG-DTG characterizations are also the useful methods to support the alkali-activation of stone wool. The phase assemblage in alkali-activated material should be performed.

Is the study design appropriate and does the work have academic merit?

Partly

Is the work clearly and accurately presented and does it cite the current literature?

Yes

If applicable, is the statistical analysis and its interpretation appropriate?

No

Are all the source data underlying the results available to ensure full reproducibility?

Partly

Are the conclusions drawn adequately supported by the results?

Partly

Are sufficient details of methods and analysis provided to allow replication by others?

Partly

Reviewer Expertise:

Geopolymer, Alkali-activated Materials, Construction Materials

I confirm that I have read this submission and believe that I have an appropriate level of expertise to confirm that it is of an acceptable scientific standard, however I have significant reservations, as outlined above.
